# Medição do Fluxo Sanguíneo Coronário em Angiogramas Coronários Convencionais por um Novo Método Baseado na Detecção da Densidade de Contraste. Uma Visão Fisiológica

**DOI:** 10.36660/abc.20180283

**Published:** 2020-09-18

**Authors:** Miguel Lopez-Hidalgo, Antonio Eblen-Zajjur

**Affiliations:** 1 Universidad de Carabobo Valencia Venezuela Universidad de Carabobo, Valencia - Venezuela; 2 Pontificia Universidad Catolica de Chile Instituto de Ingieneria Biologica y Medica Santiago Chile Pontificia Universidad Catolica de Chile - Instituto de Ingieneria Biologica y Medica, Santiago – Chile

**Keywords:** Doença da Artéria Coronariana, Síndrome Cardíaca X, Circulação Sanguínea/fisiopatologia, Angina estável, Angina Microvascular, Angiografia Coronária/métodos

## Abstract

**Fundamento:**

O grau de fluxo TIMI e a contagem quadro a quadro TIMI corrigida (CTFC) são métodos amplamente utilizados para avaliar o fluxo sanguíneo coronariano angiográfico. A medição do fluxo sanguíneo coronariano (FSC) na coronariografia (CAG) padrão despertou grande interesse recentemente, tentando combinar o conceito de CTFC com novos métodos para pós-angioplastia e avaliação da síndrome cardíaca X. Além disso, o fluxo coronariano lento é considerado um critério importante para a angina microvascular.

**Objetivo:**

Explorar uma nova abordagem de medição angiográfica quantitativa do FSC com base na detecção densitométrica de contraste na CAG offline, usando um software acessível para obter uma avaliação mais precisa e confiável do FSC.

**Métodos:**

Trinta pacientes foram estudados e divididos em 2 grupos: fluxo sanguíneo coronariano normal (FN) e fluxo sanguíneo coronariano lento (FL), de acordo com a definição da CTFC. O MD foi aplicado à amostra do estudo para diferenciar entre FN e FL. A estatística não paramétrica foi usada para avaliar diferenças entre os grupos com p<0,05.

**Resultados:**

O valor de referência normal do MD obtido para o fluxo sanguíneo coronariano foi de 9 [5–10] quadros. Os grupos FN vs. FL foi comparado e expresso como mediana [intervalo interquartil], para a artéria descendente anterior esquerda: 10 [7–11] vs. 21 [8–33]; p=0,016; artéria circunflexa: 9 [4–13] vs. 14 [11–30]; p=0,012 e artéria coronária direita: 5 [3–11] vs. 13 [8–26]; p=0,009.

**Conclusão:**

O MD mostrou a viabilidade de medir o fluxo sanguíneo coronariano com precisão, consistência e reprodutibilidade em um angiograma coronariano padrão, mostrando a capacidade adicional de diferenciar FN de FL em pacientes com dor precordial e artérias coronárias normais. (Arq Bras Cardiol. 2020; 115(3):503-512)

## Introdução

Atualmente, o método mais amplamente utilizado para avaliar o fluxo sanguíneo coronariano angiográfico na prática clínica é o sistema de graduação de fluxo TIMI – trombólise no infarto do miocárdio. As limitações de precisão e subjetividade da escala de fluxo TIMI resultaram em um método quantitativo mais preciso para avaliar objetivamente um índice de fluxo sanguíneo coronariano: contagem quadro a quadro TIMI corrigida (CTFC). A CTFC se mostrou um método mais reprodutível do que a graduação do fluxo TIMI coronariano e facilita as comparações dos desfechos angiográficos entre os ensaios.^1,2^ O desenvolvimento de um método rápido, simples e econômico para medir o fluxo sanguíneo coronariano seria de grande interesse, principalmente em pacientes com dor precordial e angiograma coronariano normal, e angina microvascular, um cenário clínico com prevalência crescente, em que o fluxo sanguíneo coronariano lento é o principal critério diagnóstico.^3^

Técnicas densitométricas para mensuração do fluxo sanguíneo coronariano já haviam sido aplicadas anteriormente, mas não foram bem-sucedidas devido a fatores técnicos, tornando sua utilização muito limitada.^1,4^ Recentemente, métodos angiográficos de medição quantitativa do fluxo sanguíneo coronariano em angiograma coronariano que não o sistema CTFC foram desenvolvidos com base em uma combinação de parâmetros anatômicos com análise coronariana quantitativa (QCA) tridimensional e dinâmica de fluidos computacional (CFD) com software dedicado que estima a avaliação funcional coronariana segmentar, atualmente validada com o padrão de referência reserva de fluxo fracionada (FFR) invasiva, entre elas: a FFR virtual (vFFR),^5^ a taxa de fluxo quantitativa (QFR), também conhecida como FFR_QCA,_^6-8^ e a FFRangio.^9,10^ Por outro lado, surgiram novas tecnologias não invasivas, como a tomografia computadorizada com múltiplos detectores e a angiografia por ressonância magnética (angio-RM), com algoritmos matemáticos complexos que detectam o fluxo sanguíneo coronariano e a reserva coronariana ainda em processo de refinamento.^11,12^ Tecnologias invasivas para a medição direta de pressões, fluxo e FFR coronarianos foram desenvolvidas e seu uso está em expansão, mas seu custo, maior invasividade e consumo de tempo do procedimento restringiram o número de serviços de hemodinâmica.^13,14^

O objetivo deste estudo foi explorar uma abordagem diferente para a medição angiográfica quantitativa do fluxo sanguíneo coronariano em um angiograma coronariano padrão (AGC), com base na detecção vídeo-densitométrica do fluxo angiográfico de contraste como substituto do fluxo sanguíneo coronariano, usando um software genérico. No presente estudo, comparamos essa abordagem com o sistema de graduação CTFC em pacientes com dor precordial e AGC normal.

## Materiais e Métodos

### Ética

O presente estudo foi aprovado pelo comitê de bioética institucional e utilizou a base de dados do serviço de hemodinâmica da instituição e os dados clínicos foram obtidos a partir do repositório do Hospital Metropolitano del Norte.

### Desenho do Estudo

Sessenta e quatro indivíduos com histórico de dor precordial submetidos a angiografia coronariana não apresentando lesões coronarianas foram selecionados inicialmente para o estudo de janeiro de 2016 a novembro de 2017, restando apenas 30 pacientes para análise final devido a critérios de exclusão, principalmente dificuldades técnicas. A amostra do estudo foi dividida usando a definição de CTFC para fluxo sanguíneo coronariano normal, em fluxo sanguíneo normal (FN) e fluxo lento (FL).

Critérios de inclusão: Pacientes >18 anos de idade, de qualquer sexo, que apresentavam dor precordial ou necessidade de angiografia urgente, que apresentavam AGC para fins diagnósticos sem lesões coronarianas, sejam estenóticas >30% ou lesões dilatadas >1,5 vezes o diâmetro normal do vaso, focal ou difuso.

Critérios de exclusão: pacientes com histórico de infarto do miocárdio, revascularização cirúrgica ou endovascular. Pacientes com cardiomiopatia dilatada, disfunção ventricular esquerda com fração de ejeção inferior a 50% por ecocardiografia 2D, cardiopatia valvular, cardiopatia congênita e doença ou anomalias coronarianas não ateroscleróticas, hipertensão resistente, cardiomiopatia hipertrófica, acidente vascular cerebral prévio, doença arterial periférica, doença renal, infecções, doença autoimune, doenças malignas e dificuldades técnicas, como AGC com gravação incompleta de imagens da fase de *washout*, ramos sobrepostos e movimentos do paciente ou da mesa que limitariam as medições densitométricas para análise.

### Desfechos e Definições

O desfecho primário foi explorar a viabilidade de medir quantitativamente o fluxo sanguíneo coronariano em um AGC pelo método densitométrico (DM) baseado na detecção densitométrica do fluxo angiográfico de contraste nas artérias coronarianas epicárdicas como substituto do fluxo sanguíneo coronariano, utilizando, pela primeira vez para esse fim, o ImageJ, um software genérico do North American National Institute of Health (NIH) para análise de imagens clínicas.

### O desfecho secundário foi avaliar a capacidade do MD de discriminar o fluxo sanguíneo coronariano normal do fluxo lento.

A determinação dos valores normais do fluxo sanguíneo coronariano pelo método densitométrico foi realizada em pacientes estáveis, utilizando AGC do o grupo de FN, tendo a CTFC como referência.^1^ De acordo com a CTFC, o fluxo sanguíneo arterial coronário normal foi definido como uma média de 21 ± 3 quadros. O fluxo coronário lento foi definido como um fluxo médio de CTFC > 2 DP limite superior a partir do fluxo normal definido ou ≥ 27 quadros. Todas as principais artérias coronárias foram avaliadas em cada paciente. O critério para inclusão dos pacientes no grupo FL foi a presença de pelo menos um vaso importante com fluxo lento.^15^ Após a obtenção da faixa normal do MD, ambos os métodos, tanto o MD quanto a CTFC, foram aplicados ao AGC de toda a amostra para avaliar a capacidade de discriminar os pacientes com FN e FL predefinidos pela CTFC.

### Coronariografia

Realizada pela técnica padrão de Judkins com administração usual de trinitrato de gliceril na dose de 75 a 100 µg.^16^ Duas projeções ortogonais ideais sem ramos sobrepostos e boa opacificação dos vasos e contraste da imagem foram selecionadas e o volume médio injetado por caso foi de 53 [42–61] ml. Todos os procedimentos foram realizados via acesso femoral, utilizando cateteres 6F. A taxa de aquisição de imagem foi de 30 quadros por segundo, resultando em uma resolução temporal de 33,3 ms. As imagens foram salvas no formato DICOM bruto e transferidas para DVD.^17^

### Processamento das Imagens

Os angiogramas coronários foram processados offline para análise densitométrica do contraste coronário em um laptop i5 executando o software ImageJ de acesso livre do NIH, v1.50i.^18^ O procedimento de medição foi realizado com a sonda digital ImageJ, que detectou densidades de fundo e contraste em uma área de 2x2 pixels quadrados, posicionada no lúmen no ponto médio entre as bordas dos vasos e nos segmentos proximal e médio de cada artéria coronária principal, começando a medição e o registro das densidades antes do aparecimento do contraste angiográfico (de fundo) e conforme sua passagem pela coronária desde a fase inicial de enchimento, passando pelo pico e após o final da fase de *washout*, quando não há mais detecção de contraste. Os valores densitométricos medianos (2x2 pixels) foram medidos para cada quadro angiográfico em unidades densitométricas arbitrárias (UDA) e expressos em uma escala de 256 níveis de cinza (preto=0 a branco=255).

Convencionalmente, os pesquisadores usaram a fase de enchimento de contraste para determinar o fluxo coronariano, como ocorre com o grau de fluxo TIMI e o sistema de CTFC. No entanto, no presente estudo, decidimos usar a fase de *washout* para melhorar a precisão e a confiabilidade do MD, com base em fatores fisiológicos que podem alterar e influenciar a real avaliação do fluxo sanguíneo coronariano durante a fase de enchimento, como a variabilidade do operador em relação ao volume, pressão e taxa de injeção manual de contraste. Considerou-se que a fase de *washout* era mais representativa e confiável do fluxo sanguíneo coronariano, porque depende absolutamente da eliminação do contraste pelo fluxo sanguíneo, o que é independente da intervenção do operador. Parte dessa abordagem exploratória é avaliar o comportamento da fase de washout para medir o fluxo sanguíneo coronariano. Embora não seja validado por outros estudos, esse parâmetro está metodologicamente relacionado à fase de preenchimento e fisiologicamente mais representativo como substituto do fluxo sanguíneo, conforme afirmado no artigo original sobre a CTFC, por Gibson et al.,^1^

Para determinar os valores de referência do fluxo sanguíneo normal, foram utilizados os pacientes estáveis e o AGC do grupo de fluxo normal. O valor de referência do MD para o fluxo coronariano normal foi calculado a partir do intervalo de tempo mediano composto dos valores densitométricos medianos da fase de *washout* de cada vaso coronariano do grupo FN, pré-especificado pela CTFC. Aplicando o fator de correção de 1,7 para a DAE, como no sistema CTFC original.

#### Etapas de obtenção dos valores de referência do fluxo sanguíneo coronariano com o método densitométrico

1. Seleção do AGC ideal de pacientes estáveis para avaliação densitométrica.
2. Classificação de AGC em FN e FL usando o sistema CTFC e selecionando o AGC do grupo de FN.
3. Execução do software ImageJ, carregamento do AGC e colocação da sonda de medição no segmento proximal do lúmen de cada artéria coronária principal.
4. Detecção da densidade de contraste, quadro a quadro, nos locais de medição.
5. Dados densitométricos salvos no formato .txt para Excel ou qualquer software estatístico (Past).
6. Traçar a curva dinâmica mediana do fluido de contraste global [25–75p] para o grupo FN de cada vaso principal.
7. A partir das curvas da etapa 6, seleção da fase de *washout* de cada vaso principal, filtragem da parte final da curva quanto ao ruído de fundo e cálculo da curva mediana da fase de *washout*.
8. Cálculo das contagens medianas compostas de quadros da fase de *washout* dos 3 principais vasos
9. Na etapa 8, o valor de referência normal do MD é obtido como um intervalo mediano e interquartil nas contagens de quadros (resolução: 33.3 ms).

#### Etapas na aplicação do método densitométrico a um paciente específico:

1. Seleção do AGC ideal para avaliação densitométrica.
2. Execução do software ImageJ, carregamento do AGC e colocação da sonda de medição de forma intraluminal no segmento proximal de cada artéria coronária epicárdica principal.
3. Detecção da densidade de contraste, quadro a quadro, nos locais de medição.
4. Dados densitométricos salvos no formato .txt para Excel ou qualquer software estatístico (Past).
5. Traçar as curvas de contraste medianas da fase de *washout*, unidades densitométricas arbitrárias (UDA) versus tempo (quadros), para cada vaso principal, filtragem da parte final da curva para minimizar o ruído de fundo.
6. Cálculo da contagem média de quadros da fase de *washout* para cada vaso principal, aplicando o fator de correção para a DAE.
7. Comparação dos valores obtidos na etapa 6 para cada vaso principal com os valores de referência do MD.
8. Classificação do AGC como FN ou FL, de acordo com os valores de referência estabelecidos pelo MD.

## Análise Estatística

As variáveis categóricas são apresentadas como contagens e porcentagens. Para detectar diferenças nas variáveis categóricas, utilizou-se o teste do qui quadrado. Os valores não paramétricos livres de distribuição da mediana e seus percentis 25–75 foram estimados para os valores densitométricos. O teste U de Mann Whitney foi utilizado para a análise das diferenças entre os grupos nas variáveis contínuas. Considerou-se um valor de p unicaudal <0,05 como estatisticamente significativo devido ao nosso interesse em detectar valores de fluxo sanguíneo lento localizado em um lado da distribuição. O coeficiente de correlação não paramétrico de Spearman (*R*) e o índice de determinação (*R*^2^) entre os valores do MD e as contagens de quadros foram calculados. Além disso, realizou-se regressão não paramétrica com uma otimização inicial de Levenberg-Marquardt, seguida pela regressão de Kriging e estimação da curva pelo modo de suavização por meio de *splines.*^19^ Os valores de referência da faixa temporal ou os critérios de corte para definir o fluxo normal com o novo MD foram feitos de forma semelhante ao método CTFC, que utilizou erro médio e padrão da contagem de quadros para cada vaso. Em vez disso, decidimos usar a faixa mediana e interquartil da curva da fase de *washout* do grupo FN pelos critérios do CTFC, excluindo os pacientes instáveis e, em seguida, calculamos a contagem de quadros densitométricos medianos e do intervalo interquartil composto das três principais artérias coronárias como um limiar para estabelecer o valor de referência para o fluxo sanguíneo coronariano normal para o MD. Foram utilizados testes não paramétricos, pois a distribuição da fase de *washout* não é normal, sendo esta a principal variável do estudo. A análise estatística foi realizada com o software Past v3.16.^19,20^

## Resultados

De um total de 64 pacientes selecionados inicialmente, sobraram 30 pacientes para o estudo, 10 no grupo FN e 20 no grupo FL. Os outros 34 pacientes foram excluídos, 29 devido a dificuldades técnicas durante a aquisição das imagens, 2 pacientes com cardiomiopatia dilatada, 2 com cardiopatias valvulares e 1 com doença autoimune (lúpus). Apenas um paciente teve aumento de troponina. Os demais não apresentavam enzimas cardíacas elevadas e seus angiogramas não apresentavam lesões obstrutivas, nem irregularidades, nem lesões de aparência difusa ou aparência de vasos finos médios e distais.

A idade mediana foi de 65 [53–67] anos. Houve maior prevalência de pacientes do sexo feminino, hipertensos e estáveis. O grupo FL apresentou maior proporção de fumantes. Setenta e três por cento dos pacientes eram clinicamente estáveis, e o procedimento foi realizado de forma eletiva. Oito pacientes foram diagnosticados com síndromes coronarianas agudas sem supradesnivelamento do segmento ST: 7 pacientes com angina instável e um com infarto do miocárdio sem supradesnivelamento do segmento ST sendo do grupo FL. Nenhum apresentava infarto do miocárdio com supradesnivelamento do segmento ST (Tabela 1). Dos 22 pacientes estáveis, 13 foram submetidos ao teste ergométrico em esteira, 7 apresentaram isquemia miocárdica, dois sendo do grupo FL.

**Tabela 1:** – Características clínicas da amostra e dos grupos pré-especificados de acordo com escala da contagem quadro a quadro TIMI corrigida do fluxo sanguíneo coronariano normal.

	Total n=30	FN: n=10 (33%)	FL n=20 (67%)	p
Idade, anos*	65 [53–67]	54 [41–67]	61[48–67]	0,33
Sexo: S	18 (60%)	7 (70%)	11 (55%)	0,34
Suspeita de DAC†	22 (73%)	6 (60%)	16 (80%)	0,20
AI/IAMSSST‡	8 (27%)	2 (20%)	6 (30%)	0,55
Hipertensão	19 (64%)	7 (70%)	12 (60%)	0,59
Dislipidemia	11 (37%)	4 (40%)	7 (35%)	0,78
Diabetes	6 (21%)	1 (10%)	5 (25%)	0,33
Tabagismo	10 (32 %)	1 (09%)	9 (45%)	0,05
Histórico familiar de DAC	6 (21%)	2 (20%)	4 (20%)	1,00
Histórico de IM	0	-	-	-

Fonte: Registro do laboratório de hemodinâmica. *Mediana [percentil 25 e 75], † DAC: Coronariopatia. AI/IAMSSST: ‡Angina instável/Infarto do miocárdio sem supradesnivelamento do segmento ST. FN: Grupo de fluxo coronariano normal; FL: Grupo de fluxo coronariano lento.

A curva dinâmica do fluido densitométrico obtida com este MD mostra uma inclinação descendente para a fase de enchimento de contraste e uma inclinação ascendente para a fase de *washout.* Seu sistema de escala densitométrica se baseia em uma escala de 256 níveis de cinza, onde o maior valor de densidade é 0 (preto=0 a branco=255).

**Figura 1:**
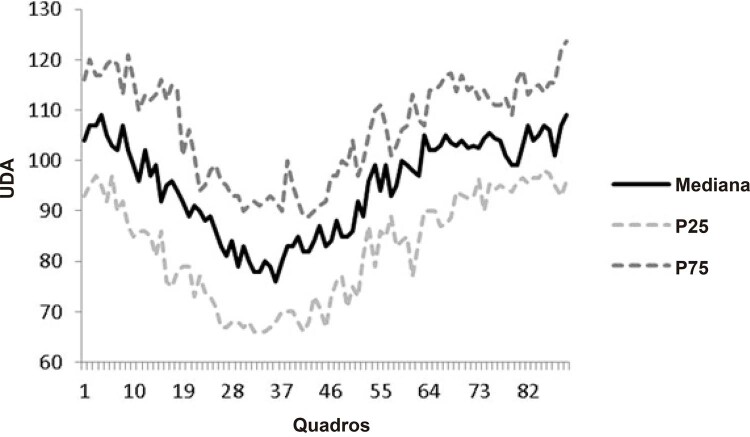
– Curva da dinâmica dos fluidos de contraste densitométrico registrada para a artéria coronária descendente anterior esquerda obtida a partir do grupo de fluxo sanguíneo normal (n=10), expressa como mediana e seus percentis 25 e 75, mostrando a curva global com uma fase de enchimento descendente e uma fase de washout ascendente.

A dinâmica dos fluidos densitométricos do contraste angiográfico que passa pelas coronárias está representada na figura 1 para a artéria descendente anterior esquerda, mostrando as fases de enchimento e *washout*; a análise dos valores medianos da fase de enchimento dos três vasos principais mostrou maior variabilidade e inconsistência, particularmente ao comparar o comportamento matemático entre os grupos FN e FL definido pelos critérios da CTFC, mostrando cruzamentos frequentes entre os dois grupos. A avaliação dos valores medianos para a fase de *washout* dos grupos FN e FL foi mais consistente. Não houve cruzamento entre os grupos, fornecendo dados mais precisos, o que confirma nossa suposição inicial. Portanto, decidimos usar a fase de *washout* para a análise.

**Figura 2:**
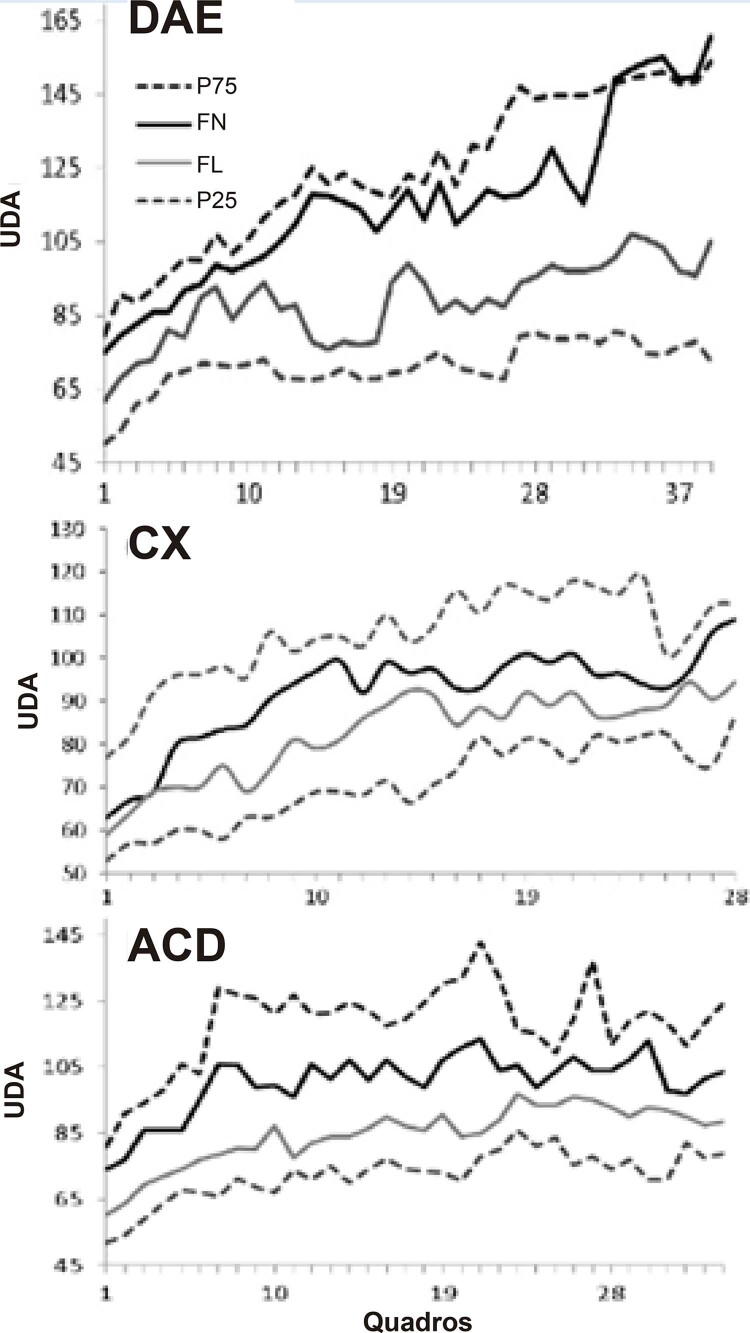
– Valores medianos e de dispersão das curvas densitométricas da fase de washout do contraste da artéria coronária descendente anterior esquerda (DAE), artéria coronária circunflexa (CX) e artéria coronária direita (ACD) para o grupo de fluxo sanguíneo normal (FN) e o grupo de fluxo sanguíneo lento (FL) após os critérios do método CTFC. P<0,0001; UDA: Unidades Densitométricas Arbitrárias. Para as equações de regressão, ver Tabela 3.

A fase mediana de *washout* mostrada como inclinações ascendentes para os grupos FN e FL é mostrada na Figura 2 para cada um dos três principais vasos, mostrando atraso estatisticamente significativo no grupo FL com a inclinação deslocada para baixo e para a direita em comparação com o grupo FN. Os dados são apresentados na tabela 2. A análise de regressão não paramétrica mostrou uma correlação positiva altamente significativa entre os valores de tempo e densidade de contraste em todas as artérias coronárias principais para os grupos FN e FL. As equações correspondentes são apresentadas na Tabela 3 para cada artéria, tanto para o grupo FN quanto para o grupo FL. Para a análise, a parte final de cada curva foi filtrada para minimizar o ruído de fundo, obtendo dados mais precisos.

**Tabela 2:** – Valores densitométricos da fase de washout dos três principais vasos coronários para o grupo de fluxo sanguíneo normal (FN) e grupo de fluxo sanguíneo lento (FL), de acordo com a definição do método CTFC, apresentados como unidades densitométricas arbitrárias (UDA) em medianas (Md) e sua faixa de percentil 25–75

**Vaso**	**FN**	**FL**	**p**
DAE UDA Md [p25–p75] Pacientes (n) Medições de quadros (n)	116 [99–122] 17 39	90 [79–90] 13 39	0,00001
CX UDA Md [p25–p75] Pacientes (n) Medições de quadros (n)	95 [86–99] 15 28	86 [74–90] 15 28	0,00025
ACD UDA Md [p25–p75] Pacientes (n) Medições de quadros (n)	104 [98–107] 16 , 30	86 [78–91] 14 30	0,00001

*DAE: Artéria descendente anterior esquerda; CX: Artéria circunflexa; ACD: Artéria coronária direita; Valor de p (teste U de Mann-Whitney).*

**Tabela 3:** – Equações de regressão não paramétrica para as artérias descendente anterior esquerda, circunflexa e coronária direita dos grupos de pacientes com fluxo sanguíneo normal (NF) ou fluxo sanguíneo lento (FL) classificados segundo os critérios do método CTFC. (UDA: Unidades Densitométricas Arbitrárias). Coeficiente de correlação de Spearman (R), coeficiente de determinação (R2) e níveis de significância (*p<0,0001)

Artéria descendente anterior esquerda:
**FN:** UDA = 109,0 exp (-((Quadros-35.5)2) / (2x12129)) R=0,85; R2 = 0,73 *
**FL:** UDA = 149.8 exp (-((Quadros-118.3)2) / (2x10973)) R=0,84; R2 = 0,70 *
**Artéria coronária circunflexa:**
**FN:** UDA = 30.6 exp (-((Quadros-120,4)2) / (2x4939,8)) R=0,80; R2= 0,64 *
**FL:** UDA = 91,4 exp (-((Quadros-22,8)2) / (2x617,38)) R=0,88; R2= 0,77 *
**Artéria coronária direita:**
**FN:** UDA = 86,9 exp (-((Quadros-30,4)2) / (2x815,9)) R=0,97; R2=0,94 *
**FL:** UDA = 85,7 exp (-((Quadros-60,0)2) / (2x3986,9)) R=0,97; R2=0,94

Comparamos os grupos FN e FL com o modelo CTFC, definido pelos critérios da CTFC para fluxo lento e, como esperado, mostrou-se que suas diferenças foram estatisticamente significantes nos dois grupos (Tabela 4).

**Tabela 4:** – Contagem quadro a quadro TIMI corrigida nos grupos FN e FL para cada vaso coronário principal, expressa em mediana e intervalo interquartil [ ], de acordo com a definição do método CTFC de fluxo normal e lento

**Vaso**	**FN n=10**	**FL n=20**	**p**
**CTFC:**	**Quadros**	**Quadros**	
**DAE**	**23 [18–26]** n=17	**44 [36–50]** n=13	0,00001
**CX**	**21 [18–28]** n=15	**41 [35–51]** n=15	0,00001
**ACD**	**23 [18–28]** n=16	**41 [33–51]** n=14	0,00001

*NF: Grupo de fluxo normal; FL: Grupo de fluxo lento; DAE: Artéria descendente anterior esquerda; CX: Artéria circunflexa; ACD: Artéria coronária direita: CTFC: Critério da contagem quadro a quadro TIMI corrigida para pacientes com fluxo coronariano lento: pelo menos um vaso principal com fluxo lento; Valor de p (teste U de Mann-Whitney).*

**Figura 3:**
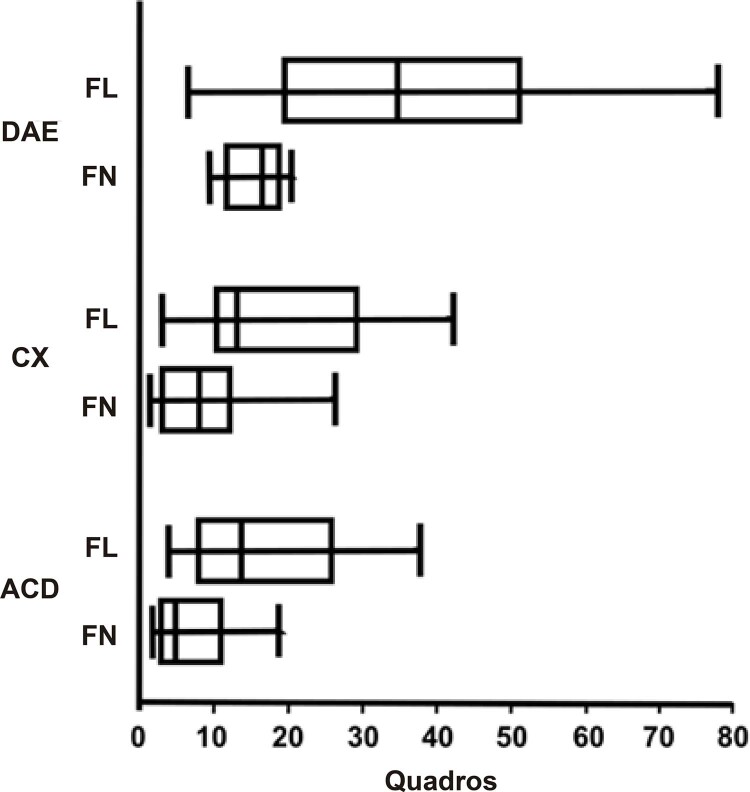
– Intervalos densitométricos medianos e intervalos interquartil para o tempo em contagens de quadros, obtidos a partir das medições densitométricas medianas da fase mediana de washout do grupo FN. Comparação entre os grupos FN e FL, pré-especificados pelo método CTFC. As diferenças são estatisticamente significantes na DAE: Artéria descendente anterior esquerda; CX: Artéria circunflexa; ACD: Artéria coronária direita; FN: Grupo de fluxo coronariano normal; FL: Grupo de fluxo coronariano lento.

O valor de referência do MD para o intervalo de tempo de fluxo coronariano normal foi de 9 [5–10] quadros (33 ms cada). Aplicando o fator de correção de 1,7 para a DAE, ou seja, dividindo o MD mediano da DAE: 16,9 por 1,7 = 10. Utilizando esse critério, a amostra de pacientes foi segregada, como FN ou FL para DAE, CX ou ACD (Tabela 5), esses grupos diagnósticos foram estatisticamente diferentes para os três vasos coronarianos, mostrando maiores valores de dispersão para FL do que os pacientes com FN (Figura 3).

**Tabela 5:** – Valores normais de referência do fluxo sanguíneo coronariano para os três principais vasos coronários pelo método densitométrico no grupo FN vs. FL, expresso em mediana [percentis 25–75], de acordo com a definição do método CTFC para fluxo normal. A DAE é suavizada por 3 pontos e corrigida pelo fator 1.7

**Vaso**	**FN**	**FL**	**p**
**DAE, n** **Quadros-MD**	16 10 [7–11]	13 21 [08–33]	0,016
**CX, n** **Quadros-MD**	14 9 [4–13]	15 14 [11–30]	0,012
**ACD, n** **Quadros-MD**	15 5 [3–11]	14 13 [8–26]	0,009

*NF: Grupo de fluxo coronariano normal; FL: Grupo de fluxo coronário lento; DAE: Artéria descendente anterior esquerda; CX: Artéria circunflexa; ACD: Artéria coronária direita; MD: Método Densitométrico; Valor de p (teste U de Mann-Whitney).*

## Discussão

Quando pacientes com suspeita de DAC cujo AGC não mostra artérias coronárias obstrutivas e aparentemente apresentam fluxo sanguíneo coronário lento, a escala de fluxo TIMI é usada para diagnosticar os fenômenos de fluxo lento, mas sendo uma medida semiquantitativa, sua precisão é baixa, principalmente para casos limítrofes. Portanto, a CTFC foi desenvolvida para uma quantificação mais precisa do fluxo sanguíneo coronariano. No entanto, uma desvantagem importante é a sobreposição entre o fluxo TIMI de grau 2 e 3, particularmente na reperfusão pos-infarto, na síndrome cardíaca X ou isquemia miocárdica e coronariopatia não-obstrutiva (INOCA), angina microvascular e nos fenômenos de fluxo lento.^1,3,21^ O desenvolvimento de novos métodos simples e práticos para a avaliação do fluxo sanguíneo coronariano na coronariografia de rotina é de extrema importância, particularmente para a avaliação da síndrome cardíaca X e da disfunção microvascular, que atualmente é um tema importante de pesquisa devido à sua significância prognóstica.^22,23^

A detecção do fluxo sanguíneo coronariano em imagens angiográficas despertou grande interesse nos últimos anos, tentando combinar o conceito de CTFC com os novos métodos de estimação da FFR a partir da coronariografia de rotina, usando uma combinação de análise coronariana quantitativa tridimensional (3D-QCA) e dinâmica computacional dos fluidos complexa.^6-8^ O uso da CTFC isoladamente não é tão prático como era no passado e a área passou a incorporar a 3D-QCA com medições de fluxo, denominada taxa quantitativa de fluxo coronariano (QFR),^6^ FFRangio^9^ estando pronta para competir com técnicas como a reserva de fluxo fracionada por tomografia computadorizada (CTFFR).^11-24^

O software ImageJ havia sido utilizado em diversas modalidades de imagem. No entanto, não havia sido utilizado anteriormente para medições hemodinâmicas. O presente estudo compara uma aplicação simples desse software validado pelo NIH com o sistema angiográfico coronário CTFC, amplamente utilizado.

Avaliamos o AGC padrão de pacientes com síndrome cardíaca X (INOCA) usando um novo método densitométrico digital, o software ImageJ, capaz de medir o fluxo sanguíneo coronariano com base na dinâmica dos fluidos de contraste que passam pelas principais artérias coronárias epicárdicas. Esse método detectou diferenças estatisticamente significativas nos intervalos de tempo do fluxo sanguíneo coronariano entre os grupos FN e FL, conforme definido pelo sistema CTFC.

A amostra do estudo apresentou maior prevalência de pacientes do sexo feminino, como visto na literatura.^3,25-27^ De acordo com alguns estudos, pacientes com fenômeno de fluxo lento coronariano apresentam características distintas de pacientes sem lesões obstrutivas angiográficas e com fluxo normal, são predominantemente do sexo masculino, fumantes e com DAC instável.^21^ No nosso estudo, observou-se maior tendência para fumantes no grupo FL, mas os outros aspectos, como sexo e apresentação instável, não diferiram do grupo FN, provavelmente devido ao tamanho da amostra e ao processo de seleção.

As técnicas densitométricas para mensuração do fluxo sanguíneo coronariano já haviam sido tentadas sem sucesso com a aplicação da densitometria, restrita às artérias coronárias proximais e não ramificadas, com traços perpendiculares ao feixe de raios X, resultando em técnicas pouco práticas.^4,28^

O MD não se baseia em referências anatômicas distais como na CTFC, tornando-o mais prático e preciso, porque essa possível variabilidade intraobservador e interobservador é eliminada da análise. Uma dificuldade frequente na CTFC é determinar o primeiro quadro inicial para a contagem, gerando um possível viés, pois depende de três critérios subjetivos de alguma forma: 1. Uma coluna de corante quase cheia ou totalmente concentrada deve se estender por toda a largura da origem da artéria; 2) O corante deve tocar as duas bordas da artéria e 3. O corante deve ter movimento anterógrado.^1^ Essa dificuldade não é contabilizada no MD. Uma vantagem importante do MD é que ele pode fornecer uma representação gráfica, o que é muito fácil de interpretar. Usando a fase de washout do contraste angiográfico em vez da fase de enchimento, nosso estudo mostra que essa poderia ser uma abordagem válida e mais confiável.

A densidade de contraste depende de vários fatores. A metodologia aplicada no presente estudo tenta reduzir a influência desses fatores na principal causa de alterações de contraste, que é o fluxo sanguíneo. As descrições originais do método CTFC^1^ minimizam a contribuição dos fatores propostos para as medições finais dos resultados, mas centralizam a discussão na correlação entre obstrução do fluxo coronariano e imagens de contraste em movimento. De fato, não houve correlação entre a CTFC de 90 minutos e a frequência cardíaca, pressão arterial sistólica ou diastólica, pressão atrial direita, diferença entre pressão arterial diastólica e pressão atrial direita, pressão de oclusão da artéria pulmonar, débito cardíaco ou índice cardíaco, mesmo após a correção da localização da artéria infartada.^1^ Outros fatores como pacientes com doença valvar aórtica, presença de fístulas, geometria anormal dos vasos ou pressão venosa central patológica foram excluídos do estudo. Fatores relevantes, como microvasculatura, e massa perfusão miocárdica, são objetivos indiretos de nossas medições.

Utilizamos o sistema de contagem quadro a quadro TIMI corrigida com base na fase de enchimento de corante de contraste das artérias coronárias como um fator de comparação, por ser a escala validada mais próxima disponível para comparação com o CTFC. No entanto, uma observação clara deve ser feita sobre as diferenças metodológicas entre a contagem quadro a quadro TIMI convencional e nossas medidas pelo MD. Escolhemos a fase de *washout* da dinâmica dos fluidos de contraste e encontramos resultados mais estáveis e constantes para a coronariografia clínica de rotina do que a fase de enchimento. Esse parâmetro está metodologicamente relacionado à fase de enchimento e, fisiologicamente, é mais representativo como substituto do fluxo sanguíneo, conforme descrito no estudo original do método CTFC, em que a eliminação do corante pode ser mais independente da taxa de injeção e merece uma investigação mais aprofundada.^1^ Nosso estudo mostra que essa poderia ser uma abordagem válida e mais confiável.

No presente estudo exploratório, os valores de referência do fluxo sanguíneo normal para o MD foram calculados usando apenas os AGCs normais do fluxo sanguíneo pelo método CTFC e em pacientes estáveis. O estudo original do método CTFC relatou valores normais de fluxo sanguíneo em pacientes submetidos a cateterismo com fluxo sanguíneo de aparência normal, sem especificar o diagnóstico ou a condição dos pacientes, com exceção do fato de que eles não apresentavam infarto do miocárdio.^1^ Para a comparação entre FN e FL, usamos toda a amostra, excluindo apenas o paciente com IAMSSST, assim como feito no estudo sobre o método CTFC e em outros estudos.^1,21^

Limitações: com o MD, todas as imagens do angiograma tiveram que ser adquiridas até a fase final de *washout,* de acordo com o que deveria ser a técnica padrão para a coronariografia; caso contrário, os dados coletados serão insuficientes para aplicar a análise densitométrica. Infelizmente, alguns operadores não realizam o procedimento de acordo com os padrões de uma técnica angiográfica adequada. É por isso que 45% da população inicial pretendida foi excluída. Este foi um estudo retrospectivo com pacientes não consecutivos e muitos pacientes foram excluídos por não atenderem aos critérios técnicos para análise do AGC. Outras limitações resultam do uso de um software genérico não dedicado que não permite a detecção de contorno e as restrições impostas às imagens bidimensionais. No entanto, essas limitações, também presentes no método CTFC convencional, não minimizaram a aplicação diagnóstica do MD descrita aqui, que foi capaz de detectar um fluxo sanguíneo coronariano confiável e lento.

O MD proposto leva cerca de 4 a 8 minutos por paciente usando um algoritmo semiautomatizado. Esse tempo pode ser drasticamente reduzido com a aplicação de um algoritmo totalmente automatizado. Estamos desenvolvendo um script ImageJ, tornando esse método mais rápido, mais prático e mais amigável. Esse novo método proposto pode ser útil se validado com uma amostra maior em um estudo prospectivo em pacientes estáveis e em outros cenários.

## Conclusões

Essa nova abordagem com o MD mostrou a viabilidade de medir o fluxo sanguíneo coronário com precisão, consistência e reprodutibilidade em um angiograma coronário padrão, mostrando a capacidade adicional de diferenciar FN e FL em pacientes com dor precordial sem obstruções coronarianas.
