# Supplementary material for: Medição do Fluxo Sanguíneo Coronário em Angiogramas Coronários Convencionais por um Novo Método Baseado na Detecção da Densidade de Contraste. Uma Visão Fisiológica
Source: Arq Bras Cardiol. 2020 Sep 18;115(3):503–12. [Article in Portuguese] doi: 10.36660/abc.20180283 (PMC9363093; doi:10.36660/abc.20180283)
Supplement: Translation

# Translation: Coronary Blood Flow Measurement in Conventional Coronary Angiograms by a New Method Based on Contrast Density Detection. A Physiological Insight

**Authors:** Miguel Lopez-Hidalgo, Antonio Eblen-Zajjur

**Affiliations:** 1 Venezuela Universidad de Carabobo, Valencia - Venezuela; 2 Chile Pontificia Universidad Catolica de Chile - Instituto de Ingieneria Biologica y Medica, Santiago – Chile

**Keywords:** Coronary Artery Disease, Cardiac syndrome X, Blood Circulation/physiopathology, Stable angina, Coronary angiography/methods, Microvascular angina

## Abstract

**Background:**

TIMI flow grade and corrected TIMI frame count (CTFC) are widely used methods to evaluate angiographic coronary blood flow. Measurement of coronary blood flow (CBF) on standard coronary angiography (CAG) has aroused great interest recently, trying to combine the CTFC concept with new methods for post-angioplasty and for cardiac syndrome X assessment. Additionally, coronary slow flow it is now considered a major criterion for microvascular angina.

**Objective:**

Explore a new approach of quantitative angiographic measurement of CBF based on densitometric contrast detection in CAG off-line, using an accessible software to obtain a more precise and reliable CBF assessment.

**Methods:**

Thirty patients were studied and divided in 2 groups, normal coronary blood flow (NF) and slow coronary blood flow (SF), according to CTFC definition. The DM was applied to the study sample to differentiate between NF and SF. Non-parametric statistics was used to assess differences between groups at p<0.05.

**Results:**

The DM normal reference value obtained for coronary blood flow was 9 [5–10] frames. NF vs SF group were compared and expressed as median [interquartile range], for the left anterior descending: 10 [7-11] vs 21 [8-33];p= 0.016; circumflex: 9 [4-13] vs 14 [11-30]; p= 0.012 and right coronary artery: 5 [3-11] vs 13 [8-26]; p=0.009.

**Conclusion:**

The DM showed the feasibility of measuring coronary blood flow with precision, consistency and reproducible in a standard coronary angiogram, showing the additional capability to differentiate between NF and SF in chest pain patients with normal coronary arteries. (Arq Bras Cardiol. 2020; 115(3):503-512)

## Introduction

Currently, the most widely use method to assess angiographic coronary blood flow in clinical practice is the thrombolysis in myocardial infarction (TIMI) flow grade scale. Precision and subjectivity limitations of the TIMI flow grade scale derived to a more precise quantitative method to objectively assess an index of coronary blood flow; this was the corrected TIMI frame count (CTFC).The CTFC showed a more reproducible method than the coronary TIMI flow grade and facilitates comparisons of angiographic end points between trials.^1,2^ The development of a fast, simple and cost-effective method to measure coronary blood flow would be of great interest, especially in patients with chest pain with normal coronary angiogram and patients with microvascular angina a clinical scenario with an increasing prevalence where coronary slow blood flow is a major diagnostic criterion.^3^

Densitometric techniques for coronary blood flow measurements had been applied before but were unsuccessful due to technical factors, making them very limited for their use.^1,4^ Recently angiographic methods of quantitative measurement of coronary blood flow on coronary angiogram other than the CTFC system had been developed based on a combination of anatomic parameters with three dimensional quantitative coronary analysis (QCA) and computational fluid dynamics (CFD) with dedicated software that estimates a segmental coronary functional assessment currently validated with the reference standard invasive coronary fractional flow reserve (FFR), among these are: the virtual FFR (vFFR),^5^ the quantitative flow ratio (QFR) also known as FFR_QCA_,^6-8^ and the FFRangio.^9,10^ On the other hand, new non-invasive technologies have emerged such as the multidetector computerized tomography and angiographic magnetic resonance imaging (angio-MRI) with complex mathematical algorithms that detects coronary blood flow and coronary reserve which are still under refinement.^11,12^ Invasive technologies for the direct measurement of coronary pressures, flow and FFR had been developed successfully and its use is expanding, but their cost, further invasiveness and time consumption of the procedure have been restricted to fewer catheterization facilities.^13,14^

The objective of this study was to explore a different approach for quantitative angiographic measurement of coronary blood flow in a standard coronary angiogram (CAG), based on video-densitometric detection of angiographic contrast flow as a surrogate of coronary blood flow, using a generic software. In the present report we compare this approach with the CTFC grading system in patients with chest pain and normal CAG.

## Materials and Methods

### Ethics

The present study was approved by the institutional bioethics committee and used the institution catheterization laboratory database and clinical data obtained from the repository of the Hospital Metropolitano del Norte.

### Study Design

Sixty-four subjects with history of chest pain who underwent coronary angiography and showed no coronary lesions were initially selected for the study from January 2016 to November 2017, only 30 patients remained for final analysis due to exclusion criteria mainly technical pitfalls. Patient sample was divided using the CTFC definition for normal coronary blood flow, into a normal blood flow (NF) and a slow flow group (SF).

Inclusion criteria: patients > 18 years old of any gender who had chest pain or the need for urgent angiography, who had CAG for diagnostic purposes without coronary lesions, either stenotic > 30% or dilated lesions > 1.5 times normal vessel diameter, focal or diffuse.

Exclusion criteria: Patients with previous myocardial infarction, revascularization either surgical or endovascular. Patients with dilated cardiomyopathy, left ventricular dysfunction with ejection fraction of less than 50 % by 2D-echocardiography, valvular heart disease, congenital heart disease and non-atherosclerotic coronary disease or anomalies, resistant hypertension, hypertrophic cardiomyopathy, previous stroke, peripheral arterial disease, kidney disease, infections, autoimmune disease, malignancy and technical pitfalls such as CAG with incomplete image saving of the washout phase, overlapping branches and patient or table motion that would limit densitometric measurements for analysis.

### End Points and Definitions

The primary end point was to explore the feasibility of measuring coronary blood flow quantitatively in a CAG, with a densitometric method (DM) based on densitometric detection of angiographic contrast flow in epicardial coronary arteries as a surrogate of coronary blood flow, using for the first time for this purpose the ImageJ software, from the North American National Institute of Health (NIH) for medical image analysis.

A secondary endpoint was to assess the capability of the DM to discriminate between normal coronary blood flow and slow flow.

Determination of normal coronary blood flow values for the densitometric method was done with stable patients using the NF group CAG taking the CTFC as a reference.^1^ According to the CTFC, normal coronary artery blood flow was defined as a mean of 21 ± 3 frames. Coronary slow flow was defined as a CTFC mean flow > 2 SD upper limit from the defined normal flow or ≥ 27 frames. All major coronary arteries were assessed in each patient. The criterion for patients inclusion in the SF group was that they had at least one major vessel with slow flow.^15^ After obtaining the DM normal range, both methods to asses the capability to discriminate between DM and the CTFC were applied to CAG of the whole sample NF and SF patients pre-defined by CTFC.

### Coronary Angiography

Performed by the standard Judkins technique with routine administration of glyceryl trinitrate at a dose of 75 to 100 µg.^16^ Two optimal orthogonal projections with no overlapping branches, good vessel opacification and image contrast were selected and the injected median volume per case was 53 [42–61] ml. All the procedures were performed via femoral access, using 6F catheters. Image acquisition rate was 30 frames per seconds, resulting in a 33.3 ms temporal resolution. The images were saved in raw DICOM format and transferred to DVD.^17^

### Image Processing

Coronary angiograms were processed off-line for coronary contrast densitometric analysis in a i5 laptop running the open access ImageJ software from NIH, v1.50i.^18^ The measurement procedure was performed using the ImageJ digital probe that detected background and contrast densities in an area of 2x2 square pixels, placed in the lumen in a midpoint between the vessel borders and at the proximal and middle segments of each major coronary artery, starting to measure and record densities before the appearance of the angiographic contrast (background) and as it passes through the coronary from the initial filling phase, the peak and after the end of the washout phase when there was no more contrast detected. Median (2x2 pixels) densitometric values were measured for every angiographic frame in densitometric arbitrary units (DAU) and expressed in a 256 gray level scale (black=0 to white=255).

Conventionally, investigators have used the contrast filling phase to determine the coronary flow, as is with the TIMI flow grade and the CTFC system. However, in the present study we decided to use the washout phase to improve the DM precision and reliability, based on physiologic factors that can alter and bias the true coronary blood flow assessment during the filling phase, such as operator’s variability related to volume, pressure and rate of manual injection of contrast. It was considered that the washout phase was more representative and reliable of coronary blood flow because it depends absolutely on the frontline of blood washing the contrast, which is independent of operator intervention. Part of this exploratory approach is to assess the behavior of the washout phase to measure the coronary blood flow. Although not validated by other studies, this parameter is methodologically closely related to the filling phase and physiologically more representative as a surrogate of blood flow, as stated in the original CTFC paper by Gibson et al.^1^

To determine the normal blood flow reference values, we used the stable patients and the normal flow group CAG. The DM reference value for normal coronary flow was calculated from the compounded median time range of the median densitometric values of washout phase of each coronary vessels from NF group, pre-specified by the CTFC. Applying the correction factor of 1.7 for the LAD, as with the original CTFC system.

#### Steps to obtain coronary blood flow reference values with the densitometric method:

1. Selection of optimal CAG of stable patients for densitometric assessment.
2. Classifying CAG for NF and SF using CTFC system, and selecting NF group CAG.
3. Running ImageJ, load the CAG and place the measurement probe at the proximal segment of the lumen of each major coronary artery.
4. Contrast density detection frame by frame at measurement sites.
5. Densitometric data saved in .txt format for excel or any statistical software (Past).
6. Plot median [25-75p] global contrast fluid dynamic curve for NF group for each major vessel.
7. From the curves in step 6, select the washout phase (WOP) of each major vessel, filter out the final portion of the curve for background noise and calculate the median WOP curve.
8. Calculate the compounded median frame counts of the WOP of the 3 major vessels.
9. From step 8, the normal DM reference value is obtained as a median and interquartile range in frame counts (resolution: 33,3 ms).

#### Steps in the application of the densitometric method to a specific patient

1. Selection of optimal CAG for densitometric assessment.
2. Running ImageJ, load the CAG and place the measurement probe intra-luminally in the proximal segment of each major epicardial coronary artery.
3. Contrast density detection frame by frame at measurement sites.
4. Densitometric data saved in .txt format for excel or any statistical software (Past).
5. Plot of the median contrast curves of the WOP, density (DAU) versus time (frames), for each major vessel, filter out the final portion of the curve to minimize background noise.
6. Calculate the median frame count of the WOP for each major vessel, applying the correction factor for the LAD.
7. Compare the obtained values in step 6 for each major vessel with the DM reference values.
8. Classification of CAG as NF or SF, according to the DM established reference values.

## Statistics

Categorical variables are presented as counts and percentages. To detect differences in categorical variables, Chi square test was used. Distribution free, non-parametrical values of median and its 25-75 percentiles were estimated for densitometric values. The Mann Whitney U test was used for the analysis of differences between groups in continuous variables. A one tailed p<0.05 was considered statistically significant due to our interest to detect slow blood flow values that is located on one side of the distribution. Non-parametric Spearman correlation coefficient (R) and the determination index (R^2^) between DM values and time frame counts were calculated. Additionally, a non-parametric regression was performed with an initial Levenberg-Marquardt optimization followed by the Kriging regression and curve estimation by its smoothing splines mode.^19^ Temporal range reference values or cut-off criteria to define normal flow with the new DM was done similarly to CTFC method which used mean and standard error of the frame count for each vessel, instead we decided to use the median and interquartile range of the washout phase curve of the NF group by the CTFC criteria excluding the unstable patients and then calculated the compounded median and interquartile range densitometric frame count of the three major coronary arteries as a threshold to establish the reference value for normal coronary blood flow for the DM. Non-parametric tests were used since the washout phase distribution is not normal, which is the main variable of the study. Statistical analysis was done with Past software v3.16.^19,2^

## Results

From a total of 64 patients initially selected, 30 patients were left for the study, 10 were in the NF and 20 in the SF group. The other thirty-four patients were excluded, 29 patients due to technical pitfalls during the acquisition of images, 2 patients had dilated cardiomyopathy, 2 valvular heart disease and 1 had autoimmune disease (lupus). Only one patient had increased troponin. The others did not have elevated cardiac enzymes and their angiograms had no obstructive lesions or irregularities, and had no diffuse appearing lesions or mid and distal fine vessel appearance.

The median age was 65 [53–67] years. There was a greater prevalence of female, hypertensives and stable patients. The SF group had a higher proportion of smokers. Seventy-three percent of the patients were clinically stable, and the procedure was performed electively and 8 were diagnosed as non-ST elevation acute coronary syndromes: 7 patients with unstable angina and one with non-ST segment elevation myocardial infarction being from the SF group, none had ST elevation MI (Table 1). From the 22 stable patients, 13 were submitted to treadmill stress test, 7 were positive for myocardial ischemia, two were from the SF group.

**Table 1:** – Clinical Characteristics of the patient sample and the pre-specified groups according to the corrected TIMI frame count scale definition of coronary normal blood flow.

	Total n=30	NF n=10 (33%)	SF n=20 67%)	p
Age, years *	65 [53–67]	54 [41–67]	61[48–67]	0.33
Sex: F	18 (60%)	7 (70%)	11 (55%)	0.34
Stable CAD †	22 (73%)	6 (60%)	16 (80%)	0.20
UA/NSTEMI ‡	8 (27%)	2 (20%)	6 (30%)	0.55
Hypertension	19 (64%)	7 (70%)	12 (60%)	0.59
Dyslipidemia	11 (37%)	4 (40%)	7 (35%)	0.78
Diabetes	6 (21%)	1 (10%)	5 (25%)	0.33
Smoking	10 (32 %)	1 (09%)	9 (45%)	0.05
CAD Family History Previous MI	6 (21%) 0	2 (20%) -	4 (20%) -	1.00 -

Source: Cath Lab Registry. * Median [25^th^ and 75^th^ percentile], † CAD: Coronary artery disease. UA/NSTEMI‡: Unstable Angina/Non-ST elevation myocardial infarction. NF: Normal coronary flow group; SF: Slow coronary flow group.

The densiometric fluid dynamic curve obtained with this DM shows a downward slope for the contrast filling phase and an upward slope for the washout phase, its densitometric scale system is based in a 256 gray level scale were the higher density value is 0, (black=0 to white=255).

The densitometric fluid dynamics of the angiographic contrast passing through the coronaries is represented in figure 1 for the left anterior descending showing the filling and washout phases, analysis of the median values for the filling phase for the 3 major vessels showed greater variability and inconsistency, particularly when comparing the mathematical behavior between NF and SF groups defined by the CTFC criteria, showing frequent cross-over between the 2 groups. Assessment of the median values for the washout phase from NF and SF groups was more consistent, there was no cross-over between the groups giving a more precise data, which confirms our initial assumption. Therefore, we decided to use the washout phase for the analysis.

**Figure 1:** – Densitometric contrast fluid dynamics curve recorded for the left anterior descending coronary obtained from the normal blood flow group (n=10), expressed as median and its 25th and 75th percentiles, showing the global curve with a downward filling phase and an upward washout phase.

The median washout phase shown as ascending slopes for the NF and SF, are shown in Figure 2 for each of the three major vessels, showing statistically significant delay in the SF group with the slope shifted downward and to the right comparing to NF group. Data is displayed in table 2. Non-parametric regression analysis showed a highly significant positive correlation between time and contrast density values in all major coronary arteries for NF and SF groups. The corresponding equations are presented in Table 3 for each artery either for NF and SF groups. For the analysis, the final portion of each curve was filtered out to minimize background noise, thus getting more precise data.

**Figure 2:** – Median and dispersion values of contrast washout phase densitometric curves of left anterior descending coronary artery (LAD), circumflex coronary artery (CX) and right coronary artery (RCA) for normal blood flow (NF) and slow blood flow (SF) groups after CTFC criteria. P < 0.0001; DAU: Densitometric Arbitrary Units. See Table 3 for regression equations.

**Table 2:** – Washout phase densitometric values of the three major coronary vessels for normal blood flow group (NF) and slow blood flow group (SF), according to the CTFC definition, presented as Densitometric arbitrary units (DAU) in medians (Md) and its 25th and 75th percentile range

**Vessel**	**NF**	**SF**	**p**
LAD DAU Md [25p-75p] Patients n Measured frames n	116 [99–122] 17 39	90 [79–90] 13 39	0.00001
CX DAU Md [25p-75p] Patients n Measured frames n	95 [86–99] 15 28	86 [74–90] 15 28	0.00025
RCA DAU Md [25p-75p] Patients n Measured frames n	104 [98-107] 16 30	86 [78-91] 14 30	0.00001

*LAD: Left anterior descending; CX: Circumflex artery; RCA: Right coronary artery; p value (Mann-Whitney U test).*

**Table 3:** – Non-parametric regression equations for left anterior descending, circumflex and right coronary arteries from normal (NF) or slow blood flow (SF) patient groups classified after CTFC criteria. (DAU: Densitometric Arbitrary Units). Spearman correlation coefficient (R), coefficient of determination (R2) and significance levels (* p < 0.0001)

Left anterior descending coronary:
**NF:** DAU = 109.0 exp (-((Frames-35.5)2) / (2x12129)) R=0.85; R2 = 0.73 *
**SF:** DAU = 149.8 exp (-((Frames-118.3)2) / (2x10973)) R=0.84; R2 = 0.70 *
**Circumflex coronary artery:**
**NF:** DAU = 30.6 exp (-((Frames-120.4)2) / (2x4939.8)) R=0.80; R2= 0.64 *
**SF:** DAU = 91.4 exp (-((Frames- 22.8)2) / (2x617.38)) R=0.88; R2= 0.77 *
**Right coronary artery:**
**NF:** DAU = 86.9 exp (-((Frames-30.4)2) / (2x815.9)) R=0.97; R2= 0.94 *
**SF:** DAU = 85.7 exp (-((Frames-60.0)2) / (2x3986.9)) R=0.97; R2= 0.94 *

We compared the NF and SF groups with the CTFC model, defined by the CTFC criteria for slow flow, and as expected, it showed that their differences were statistically significant in both groups (Table 4).

**Table 4:** – Corrected TIMI frame count in NF and SF for each major coronary vessel, expressed as median and interquartile range [ ], according to the CTFC definition of normal and slow flow

**Vessel**	**NF n=10**	**SF n=20**	**p**
**CTFC:**	**Frames**	**Frames**	
LAD	23 [18–26] n=17	44 [36–50] n=13	0.00001
CX	21 [18–28] n=15	41 [35–51] n=15	0.00001
RCA	23 [18–28] n=16	41 [33–51] n=14	0.00001

*NF: Normal flow group; SF: Slow flow group; LAD: Left anterior descending artery; CX: Circumflex artery; RCA: Right coronary artery; CTFC: Corrected TIMI frame count criterion for patients with coronary slow flow: at least one major vessel with slow flow; p value (Mann-Whitney U test).*

The DM reference value for normal coronary flow time range was 9 [5-10] frames (33 ms each) by applying the correction factor of 1.7 for the LAD; that is, dividing the LAD median DM: 16,9 by 1.7 = 10. Using this criterion, the patient sample was segregated such as NF or SF for LAD, CX or RCA (Table 5), these diagnostic groups were statistically different for the three coronary vessels, showing higher dispersion values for SF than the NF patients (Figure 3).

**Table 5:** – Coronary blood flow normal reference values for the three major coronary vessels by the densitometric method in the NF vs SF group, expressed as median [25-75 percentiles], according to the CTFC definition for normal flow. LAD its smoothed by 3 points and corrected by factor 1.7

**Vessel**	**NF**	**SF**	**p**
LAD, n Frames-DM	16 10 [7–11]	13 21 [8–33]	0.016
CX, n Frames-DM	14 9 [4–13]	15 14 [11–30]	0.012
RCA, n Frames-DM	15 5 [3–11]	14 13 [8–26]	0.009

*NF: Normal coronary flow group; SF: Slow coronary flow group; LAD: Left anterior descending artery; CX: Circumflex artery; RCA: Right coronary artery; DM: Densitometric Method; p value (Mann-Whitney U test).*

**Figure 3:** – Densitometric median and interquartile ranges for time in frame counts obtained from the median densitometric measurements of the median washout phase of the NF group. Comparison between NF and SF groups, pre-specified by the CTFC. Differences are statistically significant at LAD (p = 0.016); CX (p = 0.012) and RCA (p = 0.001; Mann-Whitney U test). LAD: Left anterior descending; CX: Circumflex artery; RCA: Right coronary artery; NF: Normal coronary flow group; SF: Slow coronary flow group.

## Discussion

When patients suspected of having CAD whose CAG shows no obstructive coronary arteries and apparently have slow coronary blood flow, the TIMI flow grade scale is used to diagnose the slow flow phenomena; but being a semi-quantitative measurement, its precision is low especially for borderline cases. Therefore, the CTFC was developed for a more precise quantification of coronary blood flow. However, an important disadvantage is the overlapping between TIMI flow grade 2 and 3 particularly in post-myocardial infarction reperfusion, in the cardiac syndrome X or myocardial ischemia and no obstructive coronary artery disease (INOCA), microvascular angina and in the slow flow phenomena.^1,3,21^ Development of new simple and practical methods for coronary blood flow assessment in routine coronary angiography is of utmost importance particularly for the evaluation of cardiac syndrome X and microvascular dysfunction which currently is a hot topic of research due to its prognostic significance.^22,23^

Detection of coronary blood flow on angiographic images has aroused great interest over the last years trying to combine the CTFC concept with the new methods of FFR estimation from routine coronary angiography using a combination of three-dimensional quantitative coronary analysis (3D-QCA) and complex computational fluid dynamics.^6-8^ Using CTFC alone is not as practical as it was in the past and the area has grown to incorporate 3D-QCA with flow measurements termed quantitative coronary flow ratio (QFR),^6^ FFRangio^9^ and is now set to compete with the likes of computer tomography fractional flow rate CT-FFR.^11-24^

ImageJ had been used in several medical imaging modalities, however, it has not previously been used for hemodynamic measurements. The present study compares a simple application of this NIH´s supported software with the widely used coronary angiographic CTFC system.

We evaluated standard CAG from patients with cardiac syndrome X (INOCA) using a new digital densitometric method, the ImageJ software, which was able to measure coronary blood flow based on contrast fluid dynamics that passes through major epicardial coronary arteries. This method detected statistically significant differences in coronary blood flow time ranges between NF and SF groups as defined by the CTFC system.

The patient sample showed a higher prevalence of female patients as seen in the literature.^3,25-27^ According to some studies, patients with coronary slow flow phenomenon have distinct features from patients without angiographic obstructive lesions and with normal flow, they are predominantly male, smokers, and with unstable CAD.^21^ Here there was a higher tendency for smokers in the SF group, but the other features such as gender and unstable presentation were not different from the NF group, probably because of the sample size and selection process.

Densitometry techniques for coronary blood flow measurements had been tried before without success applying densitometry, restricted to proximal, non-branching coronary arteries with traces perpendicular to the X ray beam, resulting in impractical techniques.^4,28^

The DM does not rely on distal anatomic landmarks as for the CTFC, making it more practical and precise because that potential intra-observer and inter-observer variability is eliminated from the analysis. A frequent pitfall in the CTFC is determining the initial first frame for counting; this gives a potential bias, because it depends in three somehow subjective criteria: 1. A column of nearly full or fully concentrated dye must extend across the entire width of the origin of the artery; 2. The dye must touch both borders of the artery, and 3. The dye must have antegrade motion,^1^ this pitfall is not accounted for in the DM. An important advantage of the DM is that it can give a graphical representation, which is very easy to interpret. Using the washout phase of angiographic contrast instead of the filling phase, our study shows that this could be a valid and more reliable approach.

The contrast density relies on several factors, the methodology applied in the present study try to reduce the influence of these factors to the main cause of contrast changes, that is blood flow. Original descriptions for the CTFC method^1^ minimize the contribution of proposed factors to the final output measurements but centers the discussion to the correlation between coronary flow obstruction and moving contrast imaging. In fact, there was no correlation between the 90-minute CTFC and heart rate, systolic or diastolic blood pressure, right atrial pressure, difference between diastolic arterial blood pressure and right atrial pressure, pulmonary capillary wedge pressure, cardiac output, or cardiac index, even after corrections for infarct-artery location was made.^1^ Other factors, such as patients with valvular aortic disease, presence of fistulas, vessel abnormal geometry or pathological central venous pressure were excluded in the study. Relevant factors such as microvasculature, myocardial mass and perfusion, are indirect aims of our measurements.

We used the corrected TIMI frame count system based on the contrast dye filling phase of coronary arteries as a comparator because it is the closest validated scale available to compare to CTFC. However, a clear remark should be made about methodological differences between conventional TIMI frame count score and our reported DM measurements. We choose the washout phase of the contrast fluid dynamics and find more stable and constant results for routine clinical coronary angiography than the filling phase, this parameter is methodologically closely related to the filling phase and physiologically more representative as a surrogate of blood flow, as clearly stated in the original CTFC report where the washout of dye may be more independent of the rate of injection and warrants further investigation.^1^ Our study shows that this could be a valid and more reliable approach.

In the present exploratory study, the normal blood flow reference values for the DM were calculated using only the normal blood flow CAGs by CTFC, and stable patients. The original CTFC study reported normal blood flow values from patients who underwent catheterization with normal appearing blood flow, without specifying the diagnosis or status of the patients, only that they didn’t had myocardial infarct.^1^ For the comparison between NF and SF we used the whole sample excluding only the patient with NSTEMI as the original CTFC and other studies did.^1,21^

Limitations: with the DM all the images of the angiogram had to be acquired until the final washout phase according to what should be the standard technique for coronary angiography, otherwise collected data will be insufficient to apply the densitometric analysis. Unfortunately, some operators do not perform the procedure according to the standards of a proper angiographic technique. That is why 45% of the initial intended population was excluded. This was a retrospective study with nonconsecutive patients and many patients were exclude because they did not comply with technical criteria for CAG analysis. Other limitations derive from the use of a non-dedicated generic software that does not allow for contour detection and the restrictions imposed on 2-D images. However, these limitations, also present on conventional CTFC, did not minimize the diagnostic application of the DM described here which was able to detect reliably slow coronary blood flow.

The proposed DM takes about 4 to 8 minutes per patient using a semi-automatized algorithm. This time can be dramatically reduced applying a fully automatized algorithm. We are developing an ImageJ script, making this method faster more practical and friendlier. This newly proposed method could prove to be useful, if validated with a greater sample in a prospective trial in stable patients and other scenarios.

## Conclusions

This new approach with the DM showed the feasibility of measuring coronary blood flow with precision, consistency and reproducible in a standard coronary angiogram, showing the additional capability to differentiate between NF and SF in chest pain patients with no coronary obstructions.
